# Effect of ECRG2 in combination with cisplatin on the proliferation and apoptosis of EC9706 cells

**DOI:** 10.3892/etm.2014.1972

**Published:** 2014-09-17

**Authors:** HAI-YAN SONG, XIAO-HUI DENG, XIN-FANG HOU, GUO-YAN YUAN, ZHEN-DONG ZHU, MING-XIN REN

**Affiliations:** 1 Key Laboratory for Medical Tissue Regeneration of Henan Province, College of Basic Medicine, Xinxiang Medical University, Xinxiang, Henan 453003, P.R. China; 2 The Affiliated Cancer Hospital of Zhengzhou University, Zhengzhou, Henan 450008, P.R. China; 3 The First Affiliated Hospital of Xinxiang Medical University, Weihui, Henan 453000, P.R. China

**Keywords:** esophageal cancer related-gene 2, bcl-2-associated X protein, cisplatin, proliferation, apoptosis

## Abstract

The aim of the present study was to explore the effect of esophageal cancer-related gene 2 (ECRG2) protein in combination with cisplatin (DDP) on the proliferation and apoptosis of esophageal cancer cells. A 3-(4, 5-dimethylthiazol-2-yl) 2,5-diphenyltetrazolium bromide (MTT) assay was used to examine the effects of ECRG2 alone and ECRG2 in combination with DDP on the proliferation of EC9706 esophageal cancer cells. Hoechst 33258 staining was performed to analyze the effects of ECRG2 alone and ECRG2 in combination with DDP on apoptosis in the EC9706 cells. The expression levels of Bcl-2-associated X protein (Bax) mRNA and protein were determined by reverse transcription polymerase chain reaction (RT-PCR) and western blot analysis, respectively. The results from the MTT assay revealed that ECRG2 inhibited the proliferation of EC9706 cells and that ECRG2 in combination with DDP had a greater inhibitory effect on cell proliferation. The antiproliferative effects were time- and concentration-dependent, within a certain range of concentrations. The Hoechst 33258 staining results demonstrated that the number of apoptotic cells following treatment with ECRG2 in combination with DDP for 24 h was higher than that following treatment with ECRG2 alone for the same duration. Western blot analysis and RT-PCR results revealed that the expression levels of Bax mRNA and protein were upregulated in cells treated with ECRG2 in combination with DDP compared with those in cells treated with ECRG2 alone. Thus, ECRG2 in combination with DDP had an enhanced inhibitory effect on EC9706 cell proliferation compared with that of ECRG2 alone, and an increased inductive effect on EC9706 cell apoptosis, possibly due to the upregulation of the expression of Bax.

## Introduction

At present, esophageal cancer has one of the highest morbidity and mortality rates, as well as one of the poorest prognosis rates, of all cancers worldwide, with >480,000 new cases and 400,000 mortalities annually (1). Only 10% of patients with esophageal cancer survive for >5 years (2). Chemotherapy is the standard regimen for patients with advanced esophageal cancer who are unable to undergo curative surgery (3). Although chemotherapy is able to improve the outcome of esophageal cancer (4,5), drug resistance and the side-effects of chemotherapy are the main reasons for therapeutic failure and the high mortality rate of esophageal cancer.

Cisplatin (DDP) is a drug that is commonly used for treatment of a number of cancers, including esophageal cancer (6). Although DDP has been used as an important agent in the treatment of esophageal cancer patients, its clinical application and efficacy have been limited due to side-effects, including neurotoxicity, hearing loss and nephrotoxicity, and the emergence of drug resistance (7).

A previous study separated and identified esophageal cancer related gene 2 (ECRG2) in normal and cancerous esophageal tissue, and hypothesized that is a tumor-suppressor gene. The results of the study revealed that the expression level of ECRG2 mRNA was lower in esophageal cancer tissue compared with the levels in esophageal tissue and a variety of other normal tissues (8). Further studies have demonstrated that ECRG2 is able to inhibit tumor cell growth and proliferation, and induce cell apoptosis *in vivo* and *in vitro* (9,10). However, to the best of our knowledge, only one study (11) has been published to date on the combined effect of DDP and ECRG2 in the treatment of esophageal cancer. Therefore, in the present study, the effects of ECRG2 in combination with DDP on EC9706 esophageal cell proliferation and apoptosis were investigated and compared with those of ECRG2 alone. The effects of the combination treatment on the expression levels of Bcl-2-associated X protein (Bax) mRNA and proteins in the EC9706 cells were also investigated.

## Materials and methods

### Materials

Human esophageal cancer cell line EC9706 was donated by the tumor cell library of the Academy of Chinese Medical Sciences (Beijing, China). The ECRG2 protein was synthesized by Shanghai Sangon Biological Engineering Technology & Services Co., Ltd (Shanghai, China). DDP was purchased from Qilu Pharmaceutical Co., Ltd (Jinan, China). The rabbit anti-human Bax and goat anti-rabbit IgG antibodies were purchased from Abcam (Cambridge, UK). Hoechst 33258 was provided by the Beyotime Institute of Biotechnology (Shanghai, China). HyClone™ RPMI-1640 medium and fetal calf serum (FCS) were obtained from GE Healthcare Life Science (Pittsburgh, PA, USA). The 3-(4,5-dimethylthiazol-2-yl)2,5-diphenyltetrazolium bromide (MTT) assay, dimethyl sulfoxide (DMSO) and TRIzol reagent were purchased from Sigma Co. (St. Louis, MO, USA). A reverse transcription and amplification kit was purchased from Promega Corporation (Madison, WI, USA).

### Cell culture

EC9706 cells were cultured in RPMI-1640 medium, containing 10% FCS (100 u/ml penicillin and 100 mg/l streptomycin), in a humidified incubator at 37°C with 5% CO_2_. Cells were in the logarithmic phase in all experiments.

### Cell viability and proliferation

In order to investigate the combined effects of DDP and ECRG2 on cell proliferation and viability, EC9706 cells (4×10^7^/l) were seeded into 96-well plates and incubated in RPMI-1640 medium supplemented with 10% FCS. Cells were randomly divided into three groups with 10 repeated wells used for each group. For the ECRG2 group, ECRG2 proteins at different concentrations (5.5, 6.5, 7.5, 8.5 μg/l) were added to the EC9706 cells. For the ECRG2 protein + DDP group, 3 mg/l DPP was added to the final concentration of ECRG2 protein, on the basis of each of the ECRG2 protein concentrations above. EC9706 cells in the control group were not treated with any drugs. Cell proliferation and viability were detected by MTT assay at 24, 48 and 72 h following treatment. To do this, 20 μl of a 5 g/l MTT solution was added to each well and incubation was continued for 4 h at 37°C. The medium was discarded and 150 μl DMSO was added to each well and agitated to fully dissolve the blue-purple MTT precipitate. A microplate reader (Bio-Rad 680; Bio-Rad, Hercules, CA, USA) was used to measure the absorbance (A) of each well at 490 nm and average values were obtained. Experiments were repeated ≥3 times and data are expressed as the mean ± standard error of the mean.

### Analysis of cell apoptosis

Hoechst 33258 staining was performed in order to investigate the rate of cell apoptosis. EC9706 cells were randomly divided into the three groups: control, ECRG2 and ECRG2 protein + DDP. For the ECRG2 group, ECRG2 proteins at different concentrations (5.5, 6.5, 7.5 and 8.5 μg/l) were respectively added to the EC9706 cells. For the ECRG2 protein + DDP groups, 3 mg/l cisplatin was added to the final concentration of ECRG2 protein, on the basis of each of the ECRG2 protein concentrations above. After 24 h, Hoechst 33258 staining was conducted for the detection of apoptosis. The apoptotic cells were observed and quantified under a fluorescence microscope (Eclipse 80i; Nikon, Tokyo, Japan) and the apoptotic rate was calculated as the ratio of the number of apoptotic cells to the total number of cells.

### Reverse transcription polymerase chain reaction (RT-PCR)

The total RNA was isolated from cells using TRIzol reagent according to the manufacturers’ instructions. The expression levels of Bax mRNA were detected by RT-PCR. The primers for Bax were 5′-TTCATCCAGGATCGAGCAGAG-3′ (forward) and 5′-TGAGGACTCCAGCCACAAAGAT-3′ (reverse). The product size was ~498 bp. The primers for glyceraldehyde 3-phosphate dehydrogenase (GAPDH) were 5′-TCATGGGTGTGAACCATGAGAA-3′ (forward) and 5′-GGCATGGACTGTGGTCATGAG-3′ (reverse). The product size was ~206 bp. Each reaction system contained 12.5 μl GoTaq^®^ GreenMaster mix (Promega Corporation, Madison, WI, USA), 2.5 μl of each primer and 5 μl cDNA. Following the activation of Taq polymerase for 5 min at 95°C, cDNA was amplified for 40 sec at 95°C, 40 sec at 55°C, and 1 min at 72°C, for 35 cycles, ending with a final extension for 5 min at 72°C. To verify the accuracy of the amplification, the RT-PCR products were electrophoresed through a 1.5% agarose gel stained with ethidium bromide. The images were collected under UV light. Data were analyzed using Light Cycler^®^ 480 software (Roche Diagnostics GmbH, Mannheim, Germany). The expression levels of mRNA were measured by densitometry. The target expressions were normalized using the expression levels of β-actin as a reference.

### Western blot analysis

The total cell proteins were extracted from the cells of the three groups. Following quantification of the total proteins, the proteins were separated through 8% polyacrylamide gel electrophoresis (PAGE) and transferred to polyvinylidene difluoride (PVDF) membranes (Millipore, Billerica, MA, USA). The membranes were blocked with 5% skimmed dried milk in phosphate-buffered saline (PBS)-Tween 20. Following incubation with a polyclonal rabbit anti-human Bax antibody (diluted 1:1,000) overnight at 4°C, the membranes were incubated with a secondary horseradish peroxidase-conjugated anti-rabbit antibody (1:1,000) for 2 h. Blots were visualized by chemiluminescence (Tanon 6200; Tanon, Shanghai, China).

### Statistical analysis

Data are expressed as mean ± standard error of the mean. Statistical analysis was performed using one-way analysis of variance on SPSS software, version 17.0 (SPSS, Inc., Chicago, IL, USA). P<0.05 was considered to indicate a statistically significant difference.

## Results

### Inhibitory effects of ECRG2 and ECRG2 in combination with DDP on the proliferation of EC9706 cells at different time points

To explore the effects of ECRG2 and ECRG2 in combination with DDP on the proliferation of EC9706 cells, an MTT assay was performed. As shown in Table I, EC9706 cell growth was inhibited by different concentrations of the ECRG2 protein in a time- and concentration-dependent manner within a certain range of concentrations. The inhibitory effect of the ECRG2 protein on EC9706 cell proliferation at different concentrations was enhanced following the addition of 3 mg/l DDP. The proliferation rate of EC9706 cells exhibited a time-and concentration-dependent reduction as the concentration of ECRG2 protein increased. The inhibition rate for the combination reached its peak (33.61%) at 72 h.

### Effect of the ECRG2 protein and DDP on the apoptosis rate of EC9706 cells

**Figure 1:**
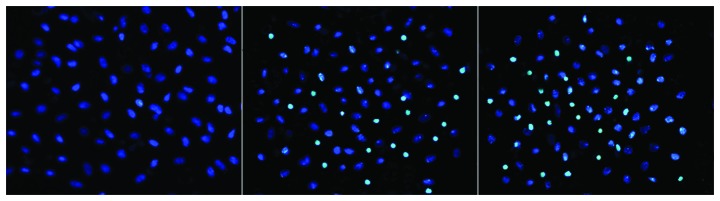
Effect of esophageal cancer-related gene 2 (ECRG2) protein and the ECRG2 protein in combination with cisplatin (DDP) on EC9706 apoptosis after 24 h. (A) Control; (B) ECRG2 protein (8.5 μg/l) and; (C) ECRG2 protein (8.5 μg/l) + DDP (3 mg/l).

As shown in Table II, the ECRG2 protein alone significantly increased the rate of EC9706 cell apoptosis compared with that of the control group in a concentration-dependent manner. When ECRG2 was used in combination with DDP, the number of apoptotic cells was significantly increased compared with that for the same concentration of ECRG2 used alone. The apoptotic effect of the combination also increased in a concentration-dependent manner. As shown in Fig. 1, the apoptotic cell bodies decreased in volume and became rounder, whilst the cell nuclei became more concentrated. Following Hoechst 33258 staining, the apoptotic cells appeared white under a fluorescence microscope.

### Effect of the ECRG2 protein and DDP on the expression levels of Bax mRNA

**Figure 2:**
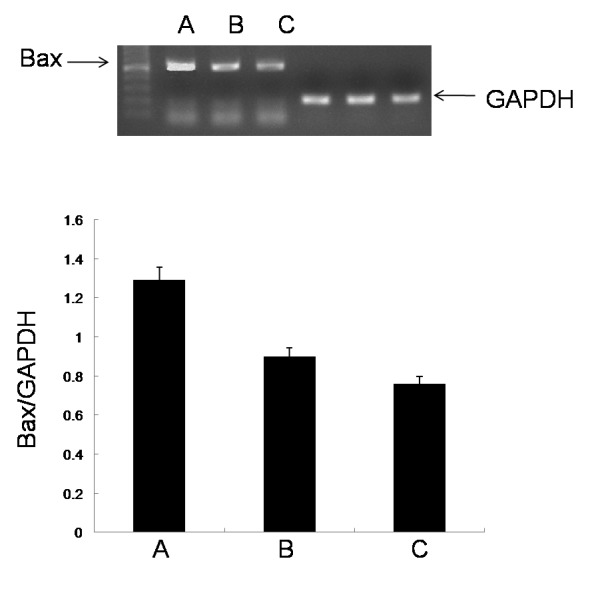
Expression levels of Bcl-2-associated X (Bax) mRNA detected by quantitative polymerase chain reaction (qPCR). (A) Esophageal cancer-related gene 2 (ECRG2) protein (8.5 μg/l) + cisplatin (DDP; 3 mg/l); (B) ECRG2 protein (8.5 μg/l) and; (C) control group. Data (n=3) are expressed as the mean ± standard error of the mean. GADPH, glyceraldehyde 3-phosphate dehydrogenase.

To investigate the mechanisms underlying the cell apoptosis induced by ECRG2 and ECRG2 in combination with DDP, the expression levels of Bax mRNA in the esophageal cancer cells were studied by RT-PCR analysis. The ECRG2 protein significantly upregulated the expression levels of Bax mRNA compared with those in the control group. When the ECRG2 protein was combined with DDP, the expression levels of Bax mRNA were significantly increased compared with those observed when the ECRG2 protein was used alone (Fig. 2).

### Effect of the ECRG2 protein and DDP on the expression levels of Bax protein

**Figure 3:**
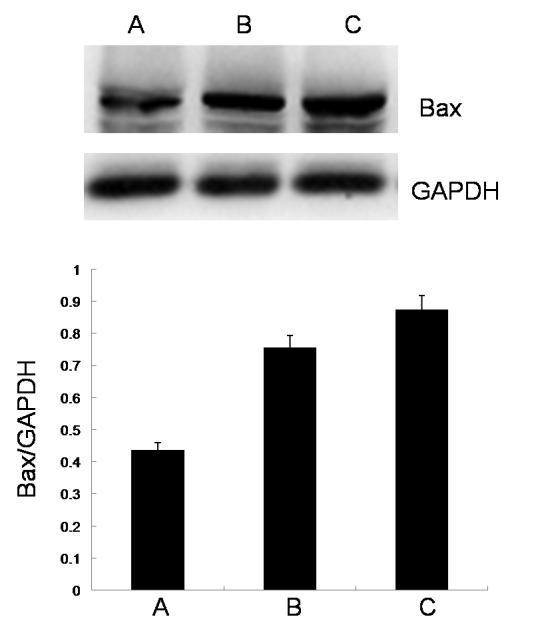
Expression levels of Bcl-2-associated X (Bax) protein by western blot analysis. (A) Control group; (B) esophageal cancer-related gene 2 (ECRG2) protein (8.5 μg/l); C: ECRG2 protein (8.5 μg/l) + cisplatin (DDP; 3 mg/l). Data (n=3) are expressed as mean ± standard error of the mean. GADPH, glyceraldehyde 3-phosphate dehydrogenase.

As shown in Fig. 3, the expression level of Bax protein was significantly upregulated by a high concentration of the ECRG2 protein compared with the level in the control group. When ECRG2 protein was combined with DDP, the expression level of Bax protein was significantly increased compared with that observed when the ECRG2 protein was used alone.

## Discussion

Esophageal cancer is one of the most common types of cancer, with one of the highest levels of morbidity and mortality, and it is a major cause of death worldwide (12). For patients with local esophageal cancer, surgical resection is the preferred treatment. However, in almost 50% of patients with esophageal cancer in clinical diagnosis, the cancer cells have already metastasized. As advanced cancer patients, these individuals mainly rely on chemotherapy for treatment. Chemotherapy is regarded as an important treatment in comprehensive therapies. DDP, a chemotherapy drug commonly used in antitumor treatments since the 1960s, destroys tumor cells mainly through the suppression of DNA replication (13); this activates apoptosis-related signaling pathways and results in cell apoptosis. However, the clinical application of DDP has been limited due to serious toxic side-effects, including neurotoxicity and loss of hearing. A method of reducing these side-effects is to decrease the dosage but leave a sufficient concentration of DDP in the blood to destroy the cancer cells. Thus, combined drug treatments have been recommended for clinical application, as they not only ensure that the concentration of platinum in the tumor tissue is sufficient to provide improved therapeutic effects, but also enable the clinical dose of the platinum-based chemotherapy drug to be reduced, which alleviates the toxic side-effects.

Human ECRG2 is located on the human chromosome 5q32–33. It comprises four exons and three introns with a total length of 3,540 bp. The ECRG2 cDNA that encodes a polypeptide with 85 amino acid residues is 569 bp in length. ECRG2 mRNA is expressed in esophageal tissue and a number of normal tissues; however, it is significantly downregulated in esophageal cancer tissue and adjacent tissues (8). ECRG2 has been revealed to inhibit tumor cell growth and proliferation, and promote cell apoptosis *in vivo* and *in vitro* (9,10,14–15). The incidence and progression of tumors results from the abnormal proliferation, differentiation and apoptosis of cells. The majority of anticancer drugs act via the induction of apoptosis in sensitive tumor cells and this drug-induced apoptotic activity of tumor cells is associated with the antitumor efficacy that is observed. Thus, the induction of apoptosis in tumor cells is a particular focus of cancer therapy studies, and apoptosis is often used as an index to evaluate the efficacy of treatments (16). In the present study, the results of Hoechst 33258 staining demonstrate that ECRG2 is able to promote the apoptosis of EC9706 esophageal cancer cells.

The Bcl-2 family are the main regulatory factors in the process of cell apoptosis through mitochondrial mediation. Bcl-2 plays an important anti-apoptotic role by preventing the release of cytochromes in the mitochondria. However, Bax has mainly pro-apoptotic effects by increasing the permeability of the mitochondrial membrane, causing the release of cytochrome *c* and subsequent cell apoptosis. A previous study revealed that cancer cells with upregulated levels of Bax exhibited a decreased tolerance to DDP (17). In another study, apoptosis of HeLa cells was induced by the upregulation of p53, which subsequently increased the expression levels of p53 and Bax (18). The apoptosis of EC9706 xenografts in mice treated with paclitaxel in a folate-mediated micelle formulation, indicated that apoptosis may be mediated via the upregulation of the expression levels of Bax (19).

EC9706 cells are a useful model in which to study the inhibition of esophageal squamous cell growth by chemical, physical and physiological agents. In the present study, using EC9706 cells as a model, when ECRG2 was combined with DDP, the inhibitory effect on cell proliferation, the inductive effect on apoptosis and the increase in the expression levels of the apoptosis-related protein Bax were significantly higher compared with those observed when the ECRG2 protein was used alone. The current results suggest that ECRG2 is not toxic and that, when used in combination with DDP, it may alleviate the toxic side-effects of DDP by allowing the clinical dosage of DDP to be reduced while still providing the desired therapeutic effect.

## Figures and Tables

**Table I:** Effect of the ECRG2 protein and ECRG2 in combination with DDP on EC9706 proliferation at different time points (%).

Group	24 h	48 h	72 h
Control	100	100	100
ECRG2 protein			
5.5 μg/l	96.24±0.62^a^	91.33±0.60^a^	87.42±0.56^a^
6.5 μg/l	92.10±0.56^a^	88.74±0.54^a^	85.40±0.60^a^
7.5 μg/l	87.39±0.52^a^	84.87±0.63^a^	80.35±0.64^a^
8.5 μg/l	81.31±0.66^a^	79.41±0.50^a^	76.10±0.57^a^
ECRG2 + DDP			
5.5 μg/l + 3 mg/l	92.48±0.40^a^,^b^	89.17±0.50^a^,^b^	85.60±0.49^a^,^b^
6.5 μg/l + 3 mg/l	88.40±0.56^a^,^b^	85.71±0.48^a^,^b^	82.15±0.60^a^,^b^
7.5 μg/l + 3 mg/l	80.84±0.54^a^,^b^	76.38±0.55^a^,^b^	73.20±0.55^a^,^b^
8.5 μg/l + 3 mg/l	74.46±0.53^a^,^b^	70.56±0.49^a^,^b^	66.39±0.50^a^,^b^

ECRG2, esophageal cancer-related gene 2; DDP, cisplatin;

a P<0.05 vs. the control group;

b P<0.05 vs. the ECRG2 group.

**Table II:** Effect of the ECRG2 protein and ECRG2 in combination with DDP on EC9706 cell apoptosis after 24 h (%).

	ECRG2 (μg/l)			
	
Group	5.5	6.5	7.5	8.5
Control	0	0	0	0
ECRG2	4.10±0.26^a^	7.20±0.25^a^	12.45±0.22^a^	18.22±0.19^a^
ECRG2 + DDP	6.40±0.21^a^,^b^	10.33±0.30^a^,^b^	16.38±0.31^a^,^b^	24.60±0.24^a^,^b^

ECRG2, esophageal cancer-related gene 2; DDP, cisplatin;

a P<0.05 vs. the control group;

b P<0.05 vs. the ECRG2 group.
